# *Lrrc7* mutant mice model developmental emotional dysregulation that can be alleviated by mGluR5 allosteric modulation

**DOI:** 10.1038/s41398-019-0580-9

**Published:** 2019-10-03

**Authors:** Chi Ho Chong, Qi Li, Priscilla Hoi Shan Mak, Cypress Chun Pong Ng, Eva Hin Wa Leung, Vicky Huiqi Tan, Anthony Kin Wang Chan, Grainne McAlonan, Siu Yuen Chan

**Affiliations:** 1 Department of Paediatrics and Adolescent Medicine, Li Ka Shing Faculty of Medicine, The University of Hong Kong, Hong Kong, China; 2 Department of Psychiatry, Li Ka Shing Faculty of Medicine, The University of Hong Kong, Hong Kong, China; 3 The Sackler Centre for Translational Neurodevelopment and The Department of Forensic and Neurodevelopmental Sciences, King’s College London, The South London and Maudsley NHS Foundation Trust, London, UK

**Keywords:** Molecular neuroscience, Neuroscience

## Abstract

*LRRC7* has been identified as a candidate gene for severe childhood emotional dysregulation. Direct experimental evidence for a role of LRRC7 in the disease is needed, as is a better understanding of its impact on neuronal structure and signaling, and hence potential treatment targets. Here, we generated and analyzed an *Lrrc7* mutant mouse line. Consistent with a critical role of LRRC7 in emotional regulation, mutant mice had inappropriate juvenile aggressive behavior and significant anxiety-like behavior and social dysfunction in adulthood. The pivotal role of mGluR5 signaling was demonstrated by rescue of behavioral defects with augmentation of mGluR5 receptor activity by 3-Cyano-*N*-(1,3-diphenyl-1*H*-pyrazol-5-yl)benzamide (CDPPB). Intra-peritoneal injection of CDPPB alleviated abnormal juvenile behavior, as well as anxiety-like behavior and hypersociability at adulthood. Furthermore, mutant primary neurons had impaired neurite outgrowth which was rescued by CDPPB treatment. In conclusion, *Lrrc7* mutant mice provide a valuable tool to model childhood emotional dysregulation and persistent mental health comorbidities. Moreover, our data highlight an important role of LRRC7 in mGluR5 signaling, which is a potential new treatment target for anxiety and social dysfunction.

## Introduction

Densin-180, also named Leucine-rich repeat containing protein 7 (LRRC7), is an abundant scaffold protein originally isolated from the postsynaptic density^1^. *LRRC7* is located at 1p31.1, a region linked to neurodevelopmental conditions including severe language impairment^2^ and autism spectrum disorder^3^. Importantly, *LRRC7* has recently been identified as a candidate gene for serious childhood emotional dysregulation. Specifically, in a genome-wide association study of the Child Behavior Checklist in children and adolescents with attention deficit-hyperactivity disorder (ADHD), SNPs in *LRRC7* were linked to the highest scores on the CBCL–DP (Child Behavior Checklist–Dysregulation Profile)^4^. High scores on CBCL–DP place an individual at an increased risk of developing serious mood disorder, anxiety, personality difficulties, and aggressive behavior which persist into adulthood^5–7^. Therefore, it is essential to understand the function of LRRC7 in brain to elucidate the molecular mechanism of the above neurodevelopmental disorders.

LRRC7 interacts with proteins such as Ca_V_1.2 channel, NMDA (*N*-methyl-d-aspartate) receptors, CaMKIIα (Ca^2+^/calmodulin-dependent protein kinase II α), δ-catenin, α-actinin, SHANK, and Maguin-1 (refs. ^8–14^). These targets have themselves been linked to a broad spectrum of neurodevelopmental disorders including autism and intellectual disability^15–22^. mGluR5 dysfunction is also associated with neurodevelopmental disorders including autism spectrum disorder and attention deficit hyperactivity disorder^23–27^. Emotional dysregulation is a common characteristic across such neurodevelopmental psychiatric disorders^28,29^. With increasing numbers of patients diagnosed with these lifelong conditions^30–32^, a better understanding of the underlying molecular mechanisms is crucial to developing effective interventions.

Although no experimental evidence suggests molecular interaction between LRRC7 and mGluR5, there have been some valuable first steps. Deletion of *Lrrc7* in C57BL/6 mice by gene targeting has been reported to reduce the level of postsynaptic mGluR5, and impaired mGluR-induced long term depression. By contrast, recruitment and function of NMDA receptors are normal at synapses^33^. However, to date, there is no direct evidence to link mGluR5 abnormalities to behavioral abnormalities in animals with *Lrrc7* deletion.

It has been well-established that mGluR5 regulates synaptic strength^34–36^, and indirect evidence shows that mGluR5 also plays a role in early neurite development. In the zebrafish model of Fragile X syndrome, pharmacological mGluR5 inhibition corrects neurite branching defects^37^; whereas in mice, deletion of mGluR5 in layer 2/3 pyramidal neurons of somatosensory cortex impairs dendritic arborization^38^. The link between mGluR5 and scaffold proteins in neurite morphogenesis and the underlying signaling remains unexplored.

Therefore, we analyzed *Lrrc7* hypomorphic mutant mice which we had fortuitously generated. We aimed to confirm a behavioral phenotype relevant to developmental emotional dysregulation. We then tested whether impaired mGluR5 signaling plays a pivotal role in causing these defects by attempting behavioral rescue through a positive allosteric modulator (PAM) of mGluR5. We tested the hypothesis that LRRC7 also enhances mGluR5-mediated neurite growth using primary embryonic neurons. Finally, we tested the prediction that augmenting mGluR5 signaling would rescue the neurite growth defects of LRRC7-deficient neurons.

## Materials and methods

### Mice

Experiments were approved by the Committee on the Use of Live Animals in Teaching and Research at the University of Hong Kong. The *Lrrc7* mutant mouse line was generated through insertional mutagenesis via microinjection of DNA into fertilized FVB/N mouse oocytes. Southern blot and inverse PCR showed insertion of a single copy of the transgene into intron 1 of *Lrrc7* gene (Supplementary Fig. S1A, B). Expression of *Lrrc7* was impaired (Fig. S1C). Western blot with antibodies for LRRC7 (Santa Cruz, cat. sc-28947) detected a trace amount of protein in homozygotes (Fig. S1D). RT-qPCR using two pairs of primers both indicated that homozygotes expressed 2% of wild-type transcripts (Fig. S1E). Primer sequences are available upon request.

We inter-crossed heterozygous mice to maintain the line. *Lrrc7* homozygous mutants appeared smaller in size since the second week of age and needed to be fed with wet chow from postnatal day (P)17 to P32, otherwise they died of dehydration. Injuries were found in littermates and recorded. A paper house was provided from birth to reduce fighting which otherwise could be fatal. Mice were weaned at around P21 but small mice were allowed to be kept with their mother until P28–P32. The weight of littermates was monitored starting from P14 to 36. At P16, the hanging wire test was used to assess motor strength before the genotype was known. The mouse was placed on the top of the cage lid. After the mouse gripped onto the wire, the lid was turned upside down. The time taken for the mouse to fall off was measured with the cut-off time set at 1 min.

### Behavioral tests

The estimation of sample size was based on the data from exploratory studies of behavior. According to PASS software (NCSS Statistical Software), eight mice should be assigned to each group to allow randomized block analysis of variance power analysis with alpha = 0.05 and power = 0.80. Mice used in behavioral experiments were littermates collected from heterozygous pairings. Mice born within the same week were employed as a cohort. Mice were tested from 8 weeks of age and tests were performed during the light cycle. The test was performed and analyzed with the genotype blinded.

The hanging wire test was used to re-assess motor strength. The time taken for the mouse to fall off from the inverted cage lid was measured. To assess behavior related to emotional dysregulation, the open field and elevated-plus maze paradigms were used to measure “anxiety”; the one-chamber and three-chamber tests were used to measure social interaction. Sucrose preference and forced swim tests were used to assess ”depression”; the Y-maze was used to examine working memory. Except for the hanging wire test and open field test, only males were employed. For the open field test, males and females behaved similarly and therefore their data were pooled.

The open field consisted of a square arena of dimension 40 × 40 × 40 cm with a marked center zone of 13.5 × 13.5 cm. The subject mouse was allowed to freely explore in the box for 10 min. Videos taken from an overhead camera were analyzed automatically using Ethovision XT7.1 (Noldus Information Technology). The data of time spent and entry to the center zone were extracted. The elevated-plus maze consisted of two opposite close arms and two opposite open arms and was elevated at a height of one meter. The subject mouse was gently placed in the center facing the close arm, and allowed to explore the maze freely for 10 min with video recording. Videos were analyzed with Ethovision XT7.1.

Wild-type males were used as strangers. In the one-chamber test, the subject mouse and a stranger mouse were introduced into a square arena of 25 × 25 × 25 cm and allowed to interact freely for 6 min. Time spent on following and sniffing the stranger mouse was scored manually from video recordings by a trained rater. The three-chamber apparatus consists of a Plexiglas box of 60 cm (L) × 40 cm (W) × 22 cm (H), divided into three chambers by transparent walls with an entry door to the middle chamber. The subject mouse was placed in the center chamber and allowed to habituate for 10 min with the doors open. Following that, it was gently moved to the center chamber and doors were closed. In one side of the chamber an unfamiliar mouse (stranger 1) was held within a wired cage, while an empty wired cage was placed in the chamber at the opposite end. The subject mouse was allowed to explore all three chambers for 10 min. Then, another unfamiliar mouse (stranger 2) was added to the wired cage in the opposite chamber. The test mouse was allowed to explore for another 10 min. Movements were video-taped and social interaction was scored manually by a trained rater.

In sucrose preference test, the subject mouse was singly caged and deprived of water the day before the test. To start (day 1), a bottle of water and a bottle of 2 % sucrose solution were provided. Both bottles were weighed every 24 h and their positions were switched to avoid position bias. Sucrose preference was measured by $\frac{{\mathrm{Volume}\,{\mathrm{of}}\,{\mathrm{sucrose}}\,{\mathrm{consumed}}}}{{{\mathrm{Total}}\,{\mathrm{volume}}\,{\mathrm{consumed}}}} \times 100{\mathrm{\% }}$ for six consecutive days. In forced swim test, a beaker of 18 cm in height and 13 cm in diameter was filled with 2 L of water and the subject mouse was gently placed inside. Their swimming time and immobile time were recorded for 6 min.

For the Y-maze test, the subject mouse was placed in a Y-shaped maze with three opaque arms at 120 ° from each other. The subject mouse was allowed to freely explore the maze for 10 min. The number of three consecutive alternations (ABC, ACB, BCA, BAC, CAB, CBA) was counted. Percentage alternation was calculated as $\frac{{\mathrm{{Number}}\,{\mathrm{of}}\,{\mathrm{3}}\,{\mathrm{consecutive}}\,{\mathrm{alternations}}}}{{{\mathrm{Total}}\,{\mathrm{number}}\,{\mathrm{of}}\,{\mathrm{arm}}\,{\mathrm{entries}}}} \times 100{\mathrm{\% }}$.

### Semi-quantitation of synaptic proteins

Cortices were dissected out and synaptosomal-enriched (P2) fraction was prepared as described^39^. Ten micrograms protein samples were loaded for western blot analysis. Proteins were detected with antibodies for mGluR5 (Abcam, cat. AB76316) and β-tubulin (Sigma, cat. T8328). Signal intensities were quantitated with ImageJ and normalized with the band intensity of β-tubulin.

### Behavioral rescue

With reference to the dosages used in other studies^24,40^, CDPPB (Tocris) was dissolved in DMSO to a concentration of 10 mg/ml, and mixed with PBS containing 10% Tween-80 in a 1:9 ratio for intraperitoneal injection. PBS containing 10% Tween-80 was given as vehicle. Mice were given a single intraperitoneal injection at 10 mg/kg/day from P16 to 22. The whole little was weaned at P35.

For rescue experiments performed in adult mice, only males were employed. CDPPB (10 mg/kg) was injected 30 min before the open field test and three-chamber test. Cortices were collected within 10 min after the open field test. Protein samples were prepared in IP buffer supplemented with inhibitors including sodium orthovanadate. Samples were probed with antibodies against phospho-ERK (Cell Signaling, cat. 9101), total ERK (Cell Signaling, cat. 9102), phospho-JNK (Cell Signaling, cat. 9251), and total JNK (Cell Signaling, cat. 9252).

### Dendrite morphology in adult hippocampus

Brains were collected from adult male littermates. Golgi-Cox staining procedures were performed following the manufacturer’s instructions (FD Neurotechnologies). Brains were sectioned coronally at 150 µm and hippocampal CA1 pyramidal neurons were reconstructed and analyzed blinded using Neurolucida software (MBF Bioscience). For comparing spine density and length, only spines in the secondary branches of apical dendrites were measured.

### Primary neuronal culture

Littermatte embryos derived from heterozygous parents were collected at day 16.5 of gestation. The hippocampus was dissected and dissociated in 0.0125% trypsin. Dissociated cells were plated at a density of 10,000 cells/cm^2^ on poly-d-lysine-coated coverslips in a 24-well plate, and cultured with neurobasal medium containing 1% B27, 2 mM GlutaMax, 25 µM l-glutamate and 1% penicillin/streptomycin for 24 h [0 day in vitro (DIV)]. Half of the medium was replaced with medium without l-glutamate on the next day.

Neurons were transfected with pEGFP (Clontech) on 5 DIV using calcium phosphate precipitation. Photomicrographs were taken on 7 DIV and analyzed blinded to genotype with the Imaris imaging software (Bitplane).

The mGluR5 antagonist 2-methyl-6-(phenylethynyl)pyridine (MPEP) (Abcam) was dissolved in PBS at 5 mM and added to culture on 3 DIV at a final concentration of 10 µM. Hippocampal neurons were transfected with pEGFP and neurite analysis was performed as described above. In rescue experiment, CDPPB (Tocris) was dissolved in DMSO (27.4 µM) and added to culture to reach a final concentration of 50 nM on 3 DIV.

### Statistical analysis

Behavioral data were collected from at least two different cohorts of mice except for one chamber test and juvenile drug rescue. Golgi analysis data were collected from three individual animals of each genotype. Morphometric analysis of primary neurons was performed on three independent experiments. Two-tailed unpaired Student *t*-test, one-way ANOVA with Tukey’s *post hoc* test, Bartlett’s test for equal variance, and two-way ANOVA with Bonferroni post hoc test were conducted using GraphPad Prism software. Pairwise comparisons of growth curves in vehicle and CDPPB-treated mice were made using SPSS. All data are represented as mean ± SEM .

## Results

### Increased anxiety and abnormal social interaction in Lrrc7 deficient mice

**Fig. 1:**
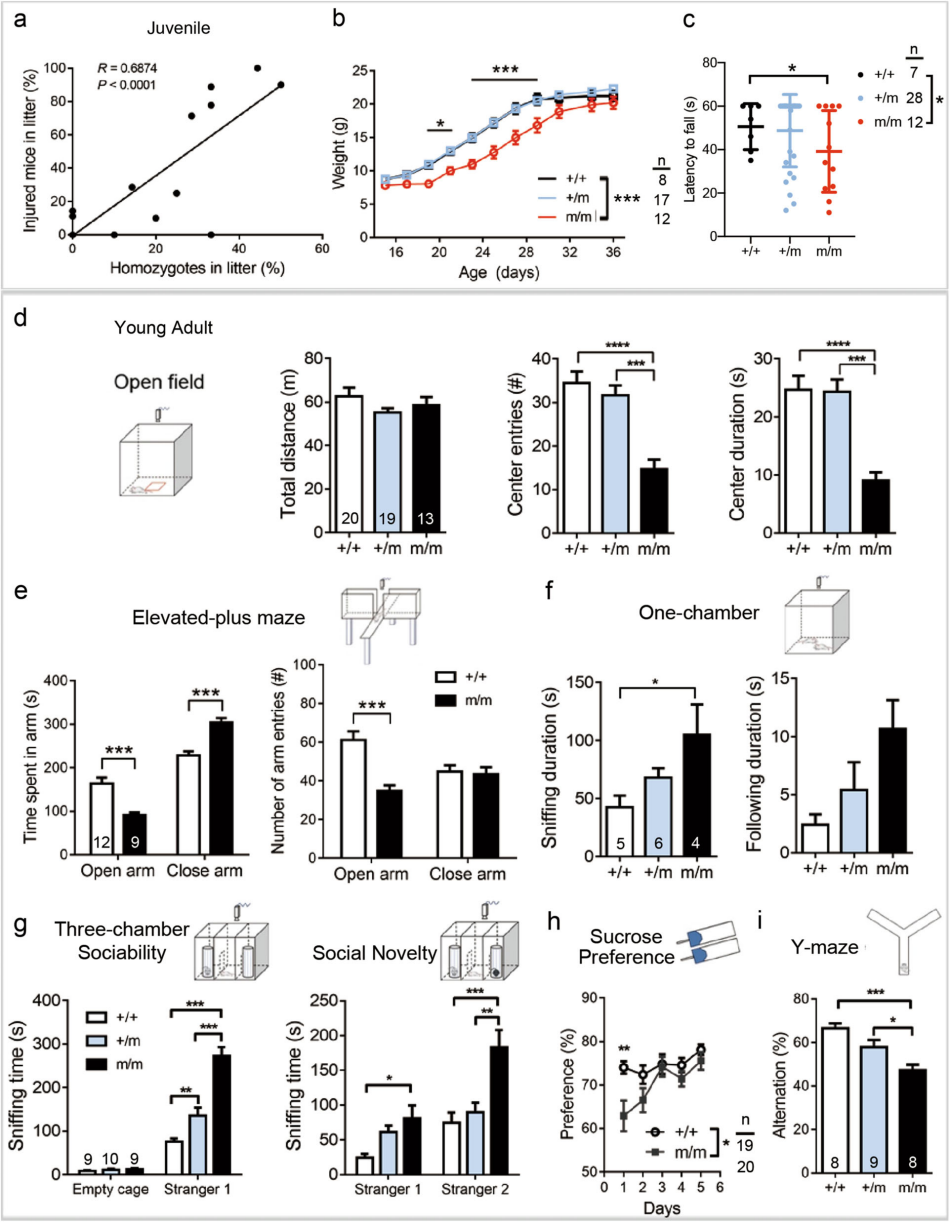
Behavioral problems relevant to emotional dysregulation in *Lrrc7* mutant mice. **a** Juvenile mutant mice caused injury to littermates as illustrated by a positive correlation between the percentage of injured mice and the percentage of homozygous mutants within the litter counted at day 26 of age. *n* = 17 litters, 8–10 mice per litter (Pearson correlation, *P* < 0.0001). **b** Transient growth retardation of homozygotes around weaning (two-way repeated measure ANOVA, genotype: *F*_(2,374)_ = 84.43, *P* = 0.00014; Bonferroni post hoc test, *P < 0.05, ***P < 0.001). **c** Wire hanging time of day 16 mi**c**e (one-way ANOVA *P* = 0.0126; Tukey’s post hoc test, *P < 0.05). Data collected from six litters. **d–i** Behavioral tests at young adulthood: **d** Mutants showed anxiety behavior in the open field test (center entries: one-way ANOVA, *P* < 0.0001; center duration: one-way ANOVA, *P* < 0.0001. Tukey’s post hoc test, ***P < 0.001, ****P < 0.0001). **e** Anxiety behavior in the elevated-plus maze as indicated by increased time spent in clos**e** arms and reduced entries to open arms (two-way ANOVA, time spent in arms: genotype: *F*_(1,38)_ = 46.69, *P* < 0.0001; arm entries, genotype: *F*_(1,38)_ = 13.06, *P* = 0.0009; Bonferroni post hoc test, ***P < 0.001). **f** Heterozygous and homozygous mice showed a trend of increased social interaction with the stranger mouse (Sni**f**fing: one-way ANOVA, *P* = 0.0399, Tukey’s post hoc test, *P < 0.05; following: one-way ANOVA, *P* = 0.0623). **g** Increased sociability of heterozygous and homozygous mutants in the three-chamber test (two-way ANOVA: Sociability, genotype: *F*_(2,40)_ = 34.90, *P* < 0.0001; Social Novelty, genotype: *F*_(2,40)_ = 13.75, *P* < 0.0001; Bonferroni post hoc test, *P < 0.05, **P < 0.01, ***P < 0.001). **h** Mutant mice showed a significantly reduced preference for sucrose solution on the first day of test (two-way repeated measure ANOVA, genotype: *F*_(1,185)_ = 6.99, *P = 0.011; Bonferroni post hoc test, **P < 0.01). **i** Impaired spatial working memory as suggested by reduced alternation in arm entry in Y-maze (one-way ANOVA, *P* = 0.003; Tukey’s post hoc test, *P < 0.05, ***P < 0.001). Number of mice indicated in graph or under *n* in diagram. +/+ wild-type; +/m heterozygotes; m/m: homozygous *Lrrc7* mutant

Mutants began to show abnormal behavior at the third to fourth week of age, regarded as the juvenile stage. Homozygotes followed their littermates excessively and this resulted in “fighting” and injuries. There was a direct correlation between the percentage of injured mice and the percentage of homozygotes in litter (Fig. 1a). Besides, homozygotes were smaller in size around weaning, but they “caught-up” at the fifth week (Fig. 1b). We checked whether mutant mice at P16 were of reduced motor strength which could affect their feeding and drinking. All tested mice were able to grip firmly and hanged for at least 11 s, but more mutant mice dropped off before the one minute cut-off time (one-way ANOVA *P* = 0.0126, Tukey’s post hoc test *P* = 0.0176 between wild-type and homozygotes; Fig. 1c).

Standard behavioral tests reported below were performed in young adults of 8–10 weeks old. Mutant mice had normal motor strength as measured by the hanging wire test at 8 weeks of age (mutant: 38.1 ± 5.5 s, *n* = 8 vs wild-type: 34.2 ± 5.4 s, *n* = 9). In the open field test, mutant mice had normal locomotion as measured by the total distance traveled. However, homozygous mutants showed markedly elevated anxiety as reflected by the significantly fewer entries to the center zone and less time spent inside the center zone (Fig. 1d). The elevated-plus maze confirmed significant “anxiety” in the homozygotes (Fig. 1e).

To evaluate social behavior of mice, one-chamber and three-chamber tests were used. No aggressive behavior of the mutant was observed in the one-chamber test. Homozygotes spent significantly more time in sniffing both in the one-chamber test (Fig. 1f) and in the three-chamber test (Fig. 1g). Compared to the wild-type, heterozygotes also spent significantly more time sniffing the stranger mouse (Fig. 1g). Thus, social interaction was disrupted by LRRC7 deficiency.

To evaluate depressive behavior, sucrose preference test was used to measure anhedonia, a core symptom in depression. Mutant mice showed a mild defect in sucrose consumption only on the first day of habituation but not the remaining 5 days (Fig. 1h), suggesting no anhedonia. In the forced swim test, mutant mice did not give up swimming throughout 6 min of the test, while the wild-type had 23 ± 9.58 s of immobility time (Student’s *t*-test, *P* < 0.0001, *n* = 6 each). Therefore, there was no evidence of depression. In the Y-maze test of spatial working memory, mutant mice showed less alternation in arm entries (Fig. 1i).

In summary, the mutant mice showed anxiety-like behavior, abnormal social behavior, signs of working memory defects but not depression-like features.

### Amelioration of behavioral defects by potentiating mGluR5 receptor signaling

**Fig. 2:**
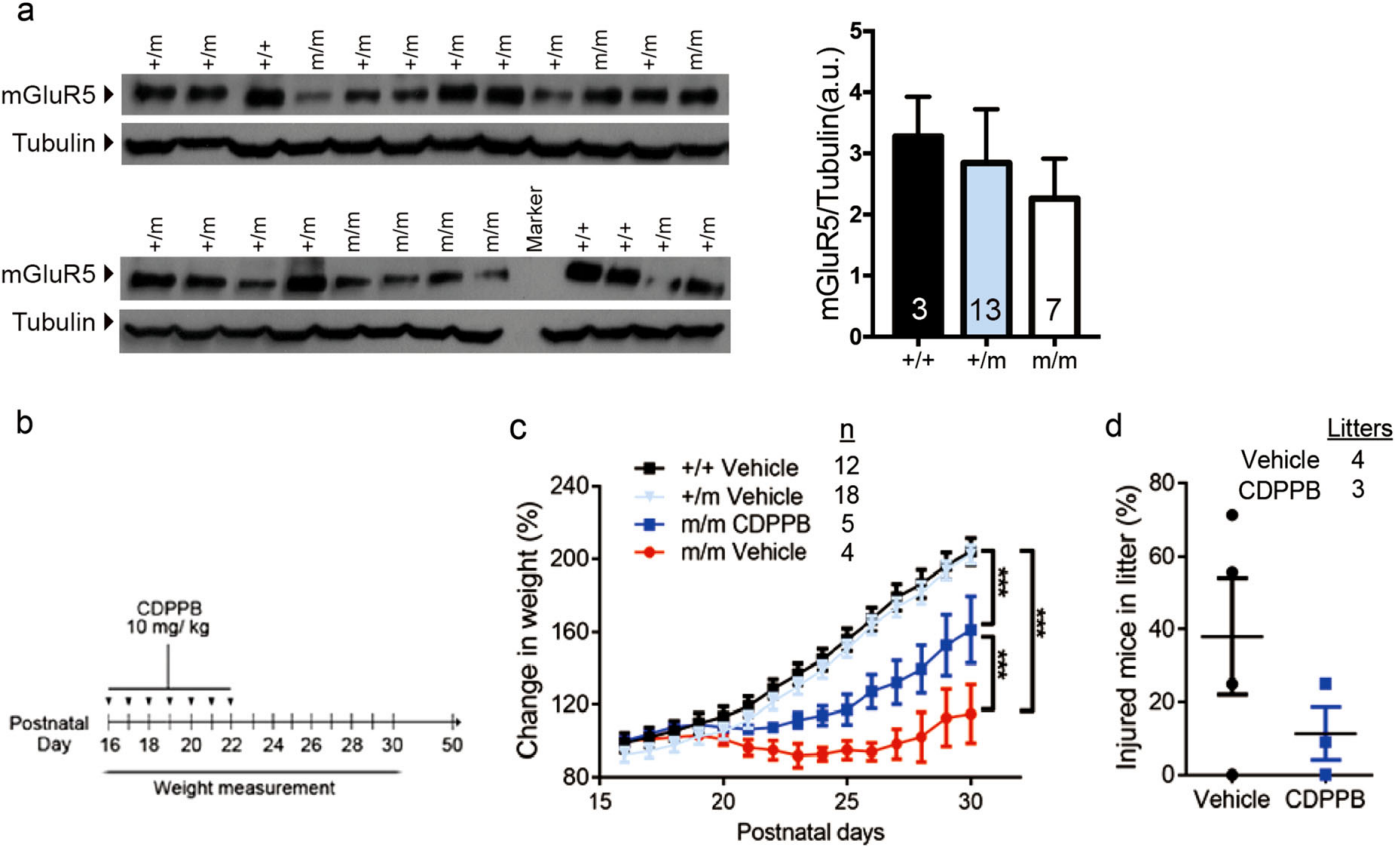
Daily intraperitoneal injection of CDPPB in juvenile mutant mice has transient beneficial effects. **a** A trend of reduced synaptic mGluR5 in mutants (one-way ANOVA, *P* = 0.1707, *n* indicated in graph). **b** Schematic diagram of the CDPPB treatment protocol. The whole litter of mice from heterozygous breeding pairs was kept with their mother throughout the experiment. **c** Homozygous mutant mice had significantly better weight gain after drug treatment (two-way repeated measure ANOVA, group: *F*_(3,490)_ = 6.59, *P* = 0.0012; no significant difference between +/+ and +/m. *P* < 0.001 for all other pairwise comparison between two groups. Number of mice (*n*) indicated in graph. Data collected from seven litters. **d** Same mice showed a trend of reduced injury when counted at postnatal day 26 in CDPPB-treated litters (*P* = 0.236). +/+, wild-type; +/m, heterozygotes; m/m, homozygous *Lrrc7* mutant

Since we detected a near significant reduction in the expression of mGluR5 in synaptosome-enriched fraction of brain lysates of mutants collected at P28 (Fig. S2), we moved on to test the beneficial effect of augmenting mGluR5 signaling by using a PAM that can cross the blood brain barrier^41^. We confirmed that at P16, there was a reduction in the synaptosomal level of mGluR5 in some of the mutants but overall there was no significant difference between genotypes (Fig. 2a). As homozygous mutant mice were profoundly smaller than littermates at days 24 to 28, we tested whether daily injection of CDPPB from P16 to 22 could be preventive (Fig. 2b). The result showed that mutant mice were still below normal weight at P28 to P30, but they had significantly better weight gain compared to vehicle injected mutants (Fig. 2c). Furthermore, fighting and injuries were less apparent on P26 (Fig. 2d). This effect did not persist and more injured mice were observed 2 days later and mutant mice showed anxiety-like behavior in the open field test at 8 weeks (Fig. S3).

**Fig. 3:**
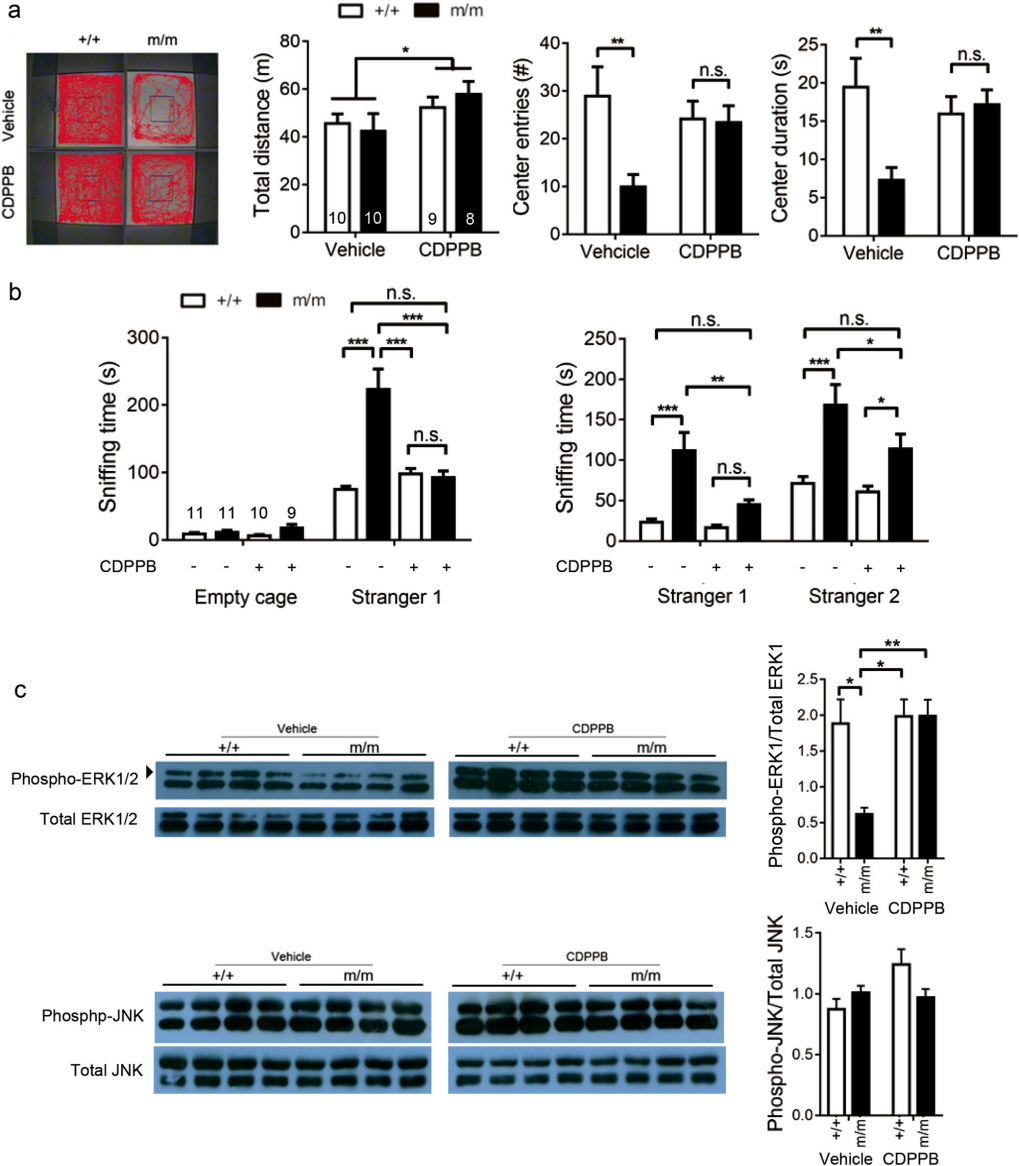
Acute injection of CDPPB alleviates the anxiety-like behavior and excessive social interaction in mutant mice. **a** Injection of CDPPB 30 min prior to the test restored normal behavior of mutant mice in the open field. Moving tracks of mice and parameters in 10 min were shown (two-way ANOVA: total distance, drug: *F*_(1,33)_ = 4.24, *P* = 0.0475; center entries, genotype: *F*_(1,33)_ = 4.99, *P* = 0.0324; center duration, genotype: *F*_(1,33)_ = 4.19, *P* = 0.0487). **b** CDPPB alleviated hypersociability in mutant mice in the three-chamber test (two-way ANOVA: Sociability—stranger 1, genotype: *F*_(1,37)_ = 16.50, *P* = 0.0002, drug: *F*_(1,37)_ = 9.37, *P* = 0.0041; Social novelty—stranger 1, genotype: *F*_(1,37)_ = 20.02, *P* < 0.0001, drug: *F*_(1,37)_ = 8.05, *P* < 0.0074; Social novelty—stranger 2, genotype: *F*_(1,37)_ = 19.38, *P* < 0.0001, drug: *F*_(1,37)_ = 3.61, *P* = 0.0651) (a, b, Bonferroni post hoc test *P < 0.05, **P < 0.01, ***P < 0.001; n.s., not significant. Number of mice indicated in graph on the left). **c** Normal JNK but impaired ERK1 signaling (arrow) in the mutant cortex. Protein lysates were collected within 10 min after the open field test. Quantitation of western blot shown on the right. *P < 0.05, **P < 0.01, Student *t*-test; +/+, wild-type; m/m, homozygous *Lrrc7* mutant

To further explore the therapeutic potential of CDPPB, we tested if acute injection of the drug in treatment naive mice could ameliorate anxiety-like behavior and hypersociability. Injection of CDPPB 30 min before the open field test or three-chamber test eliminated the difference between wild-type and homozygous mutants in terms of the number of center entries and the duration in the center (Fig. 3a); CDPPB also restored the sniffing time of the mutants to normal in the three-chamber test when only stranger 1 was present (Fig. 3b, left). When stranger 2 was introduced, there was also significant reduction in sniffing time both toward strangers 1 and 2 (Fig. 3b, right).

Brain lysates were collected from cortices right after open field test for investigating the effect of CDPPB injection on known mGluR5 downstream signaling pathways^42^. Without CDPPB treatment, we detected impaired phosphorylation of ERK, but not JNK, in mutant cortices. Mice treated with CDPPB 30 min before the test did not show this difference (Fig. 3c).

### Lrrc7 is important in dendrite morphogenesis

**Fig. 4:**
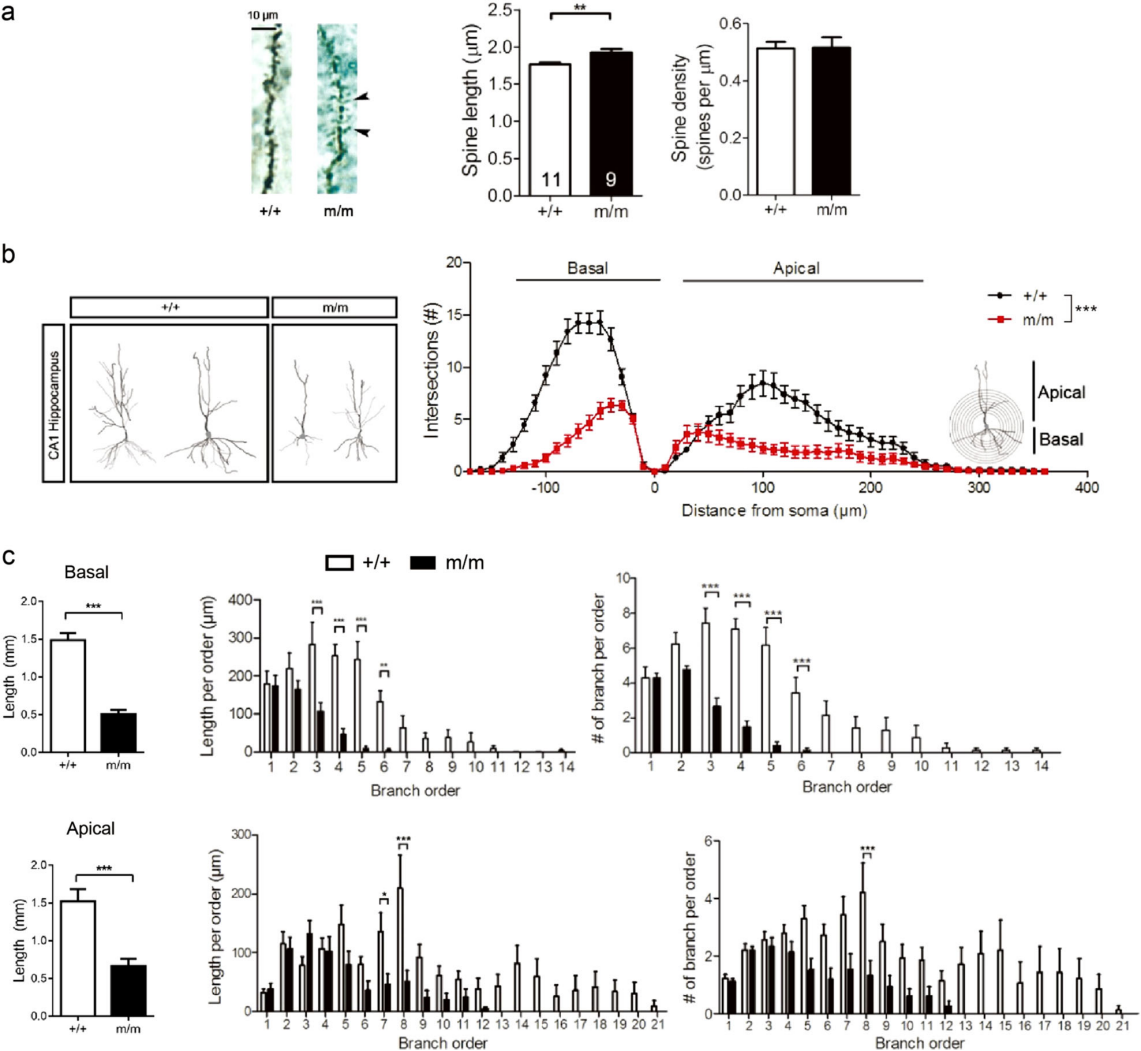
Deficiency in LRRC7 causes reduced dendritic arborization in the hippocampus. **a** Mutant pyramidal neurons in the hippocampus showed elongated dendritic spines (arrows). Secondary apical dendrites of neurons from the CA1 region were analyzed by Neurolucida software. Student's *t*-test, **P < 0.01. No. of dendrites analyzed indicated in graph; three mice per genotype. **b** Reduced dendritic complexity in mutant hippocampal neurons as shown by Sholl analysis (Genotype, two-way ANOVA: *F*(1, 864) = 309.5, ***P < 0.0001**)**. **c** Reduced length and branching in both basal and apical dendrites in mutant pyramidal neurons as analyzed by Neurolucida software (Basal: total length, Student's *t*-test, ***P < 0.001; length per order, two-way ANOVA: genotype: *F*(1, 112) = 50.42, *P* < 0.0001; number of branch per order, genotype: *F*(1, 112) = 94.77, *P* < 0.0001; apical: total length, Student's *t*-test, ***P < 0.001; length per order, two-way ANOVA: genotype: *F*(1, 240) = 17, *P* < 0.0001; number of branch per order, genotype: *F*(1,240) = 33.21, *P* < 0.0001). Bonferroni post hoc test, *P < 0.05, **P < 0.01, ***P < 0.001. Number of neurons in **b** and **c**: +/+, wild-type = 14; m/m, mutant = 15 from three mice each

The in vivo significance of LRRC7 in dendritic branching is not well established. Therefore, we analyzed the morphology of Golgi-stained pyramidal neurons from the CA1 region of adult hippocampus and compared between littermates. Mutant neurons had significantly elongated spine neck (Fig. 4a). Moreover, Sholl analysis of the 3D reconstructed CA1 neurons revealed that mutant neurons were less complex than wild-type (Fig. 4b). Specifically, mutants had significantly reduced length and branching starting at the third order for basal dendrites. For the apical dendrites, this occurred at the fifth order of branching and became significant at the seventh and eighth order (Fig. 4c).

### LRRC7 regulates neurite morphogenesis via mGluR5 signaling

**Fig. 5:**
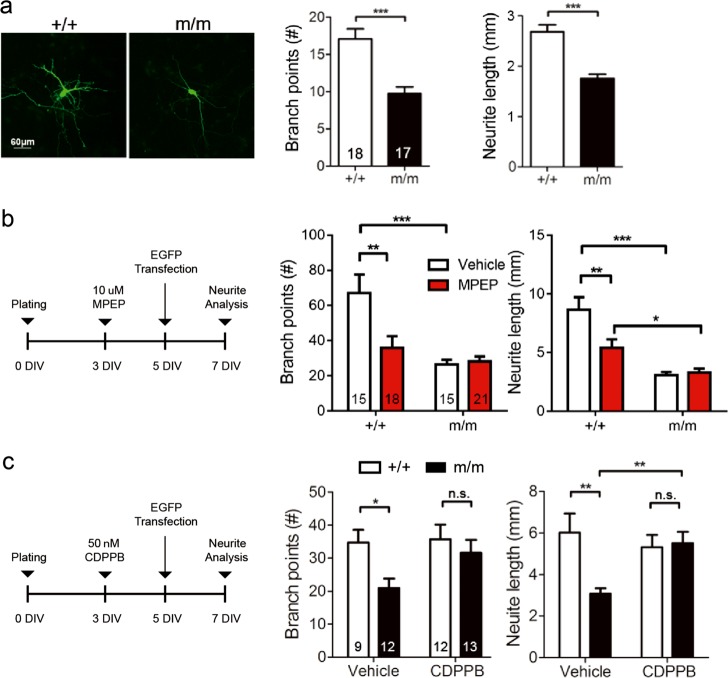
LRRC7 enhances mGluR5 signaling in neurite growth. **a** EGFP transfected hippocampal neurons were measured at 7 DIV. Mutant neurons showed reduced neurite growth. Student's *t*-test, ***P < 0.001. **b** mGluR5 specific antagonist MPEP reduced branch number and length in wild-type neurons, but did not further reduce neurite growth in mutant neurons (two-way ANOVA: branch points, drug: *F*_(1,63)_ = 7.99, *P* = 0.0063; length, drug: *F*_(1,63)_ = 8.72, *P* = 0.0044, Bonferroni post hoc test, *P < 0.05, **P < 0.01, ***P < 0.001). **c** Addition of CDPPB rescued the neurite growth defect in mutant neurons (two-way ANOVA: branch points, genotype: *F*_(1,42)_ = 5.55, *P* = 0.0232; length, genotype: *F*_(1,42)_ = 5.68, *P* = 0.0218). Bonferroni post hoc test, *P < 0.05, **P < 0.01, n.s., not significant. Number of neurons as indicated in bars. Neurons collected from three independent experiments. +/+, wild-type; m/m, homozygous *Lrrc7* mutant

As we found that neurite morphogenesis was significantly impaired in mutants, we used primary neuronal cultures to test whether LRRC7-mGluR5 directly controls this process. Primary hippocampal neurons were transfected with EGFP plasmid at 5 DIV for accurate tracing of neurites at 7 DIV. Similar to ex vivo tissues from adult animals, neuron morphogenesis was impaired in mutants (Fig. 5a). To validate that mGluR5 signaling is important for neurite development, we treated neurons with the selective mGluR5 antagonist MPEP. The treatment reduced the total number of branch points and the total neurite length in wild-type neurons, but did not further reduce neurite growth in mutant neurons (Fig. 5b). Finally, we tested whether neurite growth could respond to CDPPB treatment. We applied CDPPB to the cultures since 3 DIV, transfected with EGFP at 5 DIV and measured neurite growth 2 days later. With CDPPB, both the number of branch points and neurite length were restored in the mutant (Fig. 5c).

## Discussion

The importance of scaffold proteins in synapse development and hence neurodevelopmental conditions such as ADHD, autism spectrum disorder, bipolar disorder, and schizophrenia is increasingly recognized^20,24,43–45^. Here we report that disruption of the *Lrrc7* gene, which has been linked to serious childhood emotional dysregulation^4^, impairs signaling of mGluR5. The functional consequences comprise anxiety and social interaction impairments analogous to the difficulties reported in individuals with neurodevelopmental conditions. In addition, we provide the first evidence that both behavioral and cellular phenotypes of these mutant animals can be rescued by CDPPB, confirming a crucial role for mGluR5 signaling in this model of emotional dysregulation. Using primary hippocampal neurons to examine neurite growth, we also established a new role of LRRC7–mGluR5 signaling in neurite aborization.

In mice, an *Lrrc7* loss-of-function mutant line on the C57BL/6 genetic background has been reported to be small at weaning and to develop anxiety, excessive fighting, impaired short-term memory, and disrupted sensorimotor gating^33^. Here, we found that our mutant mice were also small around the time of weaning, and had anxiety-like behavior. However, in our studies, the rearing conditions also affected the phenotypic manifestation. We provided a paper house in the cage since this practice reduced fighting and fatal attacks. We also analyzed the social interaction of the mutant mice with strangers and found hypersociability in mutant mice, with heterozygotes showing a milder phenotype than homozygotes. For anxiety-like behavior, even though the heterozygous mice behaved normally in the open field test, we found in the behavioral rescue experiment that stress at the juvenile stage could lead to anxiety-like behavior in adulthood (Supplementary Fig. S3). Taken together, reduced expression of *Lrrc7* had a profound impact on anxiety and social behavior on different genetic backgrounds, with manifestation influenced by rearing conditions.

It is important to test whether early drug intervention could be beneficial or even preventive in developmental disorders. In particular, adolescent administration of mGluR5 PAMs can prevent adult-onset deficits induced by neonatal treatment with phencyclidine^40^; and 1 week administration with a mGluR5 PAM attenuated sensorimotor gating deficits in a similar model^46^. When CDPPB was given for 7 days at juvenile stage in our mouse model, it was effective in improving weight gain and reducing juvenile fighting. Furthermore, a single injection of CDPPB in adult mutants significantly alleviated the anxiety-like behavior in the open field test and hypersociability in the three-chamber test. Thus, although LRRC7 controls neuron morphogenesis since early development, the effects of its loss are not completely irreversible in the mature brain. This is very promising and fits with other preclinical studies that mGluR5 may be a tractable drug target. For example, acute CDPPB injection is effective in alleviating abnormal behaviors in rodent models of schizophrenia^41^, tuberose sclerosis^24^, and autism spectrum disorder^47^. Augmentation of signaling by PAMs instead of mGluR agonists has the potential advantage of high receptor subtype selectivity and low receptor desensitization^48–50^; PAMs also maintain the intrinsic regulatory systems for neurotransmitter release^48–50^. Furthermore, improved mGluR5 PAMs have been developed including one which is effective when given orally^51^.

New treatment targets for socio-emotional disorders across the life-span are much needed. In clinical settings, treatments targeting mGluR5 are starting to be investigated. These include AFQ056, an mGluR5 antagonist, which is being trialed in people with obsessive compulsive disorder who are resistant to selective serotonin reuptake inhibitors (Trial no. NCT01813019). Especially in children, the need for safer and more effective interventions for serious mood and emotion difficulties is most marked because medications such as selective serotonin reuptake inhibitors, commonly used in adults^52^, are not ideal for children^53^. In adolescents with ADHD and glutamatergic gene network variants, fasoracetam, which activates mGluRs, has completed Phase 1 trial^54^. We suggest *LRRC7* genotyping could be incorporated in future patient stratification. This group of patients will potentially benefit from mGluR5 PAMs or drugs such as fasoracetam.

The detailed mechanism of how LRRC7 affects mGluR5 signaling and how CDPPB alleviates the behavioral defects remain to be established. In the synapse, one proposed mechanism is LRRC7 indirectly affects the localization of mGluR5 through reducing the amount of α-actinin, a common interacting protein of mGluR5 and LRRC7 (ref. ^33^). For repeated treatment, there are also indirect effects of mGluR5 PAMs on the expression of NMDA receptors in specific brain regions in rat models^55^.

In conclusion, *Lrrc7* mutant mice may provide a valuable tool to model developmental emotional dysregulation and persistent mental health difficulties. The model has allowed us to pinpoint the importance of LRRC7–mGluR5 pathway in behavioral control. Our data highlight the acute effect of mGluR5 PAM in treatment of anxiety and socio-emotional disorders. Issues such as treatment duration and dosage responses, as well as the long-term outcome have to be resolved. Furthermore, both female and male mutant mice were affected but we used males only in the social interaction tests and the rescue experiments. Given the possibility of different pharmacological responses to drugs in males and females^56^, a comprehensive study in both sexes is warranted. Detailed mechanistic study on how the mature but elongated spines restructure and function, and how they response to drug treatment will provide further insights into the role of scaffold protein in signaling and synaptic plasticity. We hope this work will encourage further translational studies in the clinical setting.

## Supplementary information

Supplementary Figs
