# Supplementary material for: Lrrc7 mutant mice model developmental emotional dysregulation that can be alleviated by mGluR5 allosteric modulation
Source: Transl Psychiatry. 2019 Oct 3;9:244. doi: 10.1038/s41398-019-0580-9 (PMC6776540; doi:10.1038/s41398-019-0580-9)
Supplement: Supplementary file 1 — Supplementary Figs [file 41398_2019_580_MOESM1_ESM.docx]

***Lrrc7* mutant mice model developmental emotional dysregulation that can be alleviated by mGluR5 allosteric modulation**

**
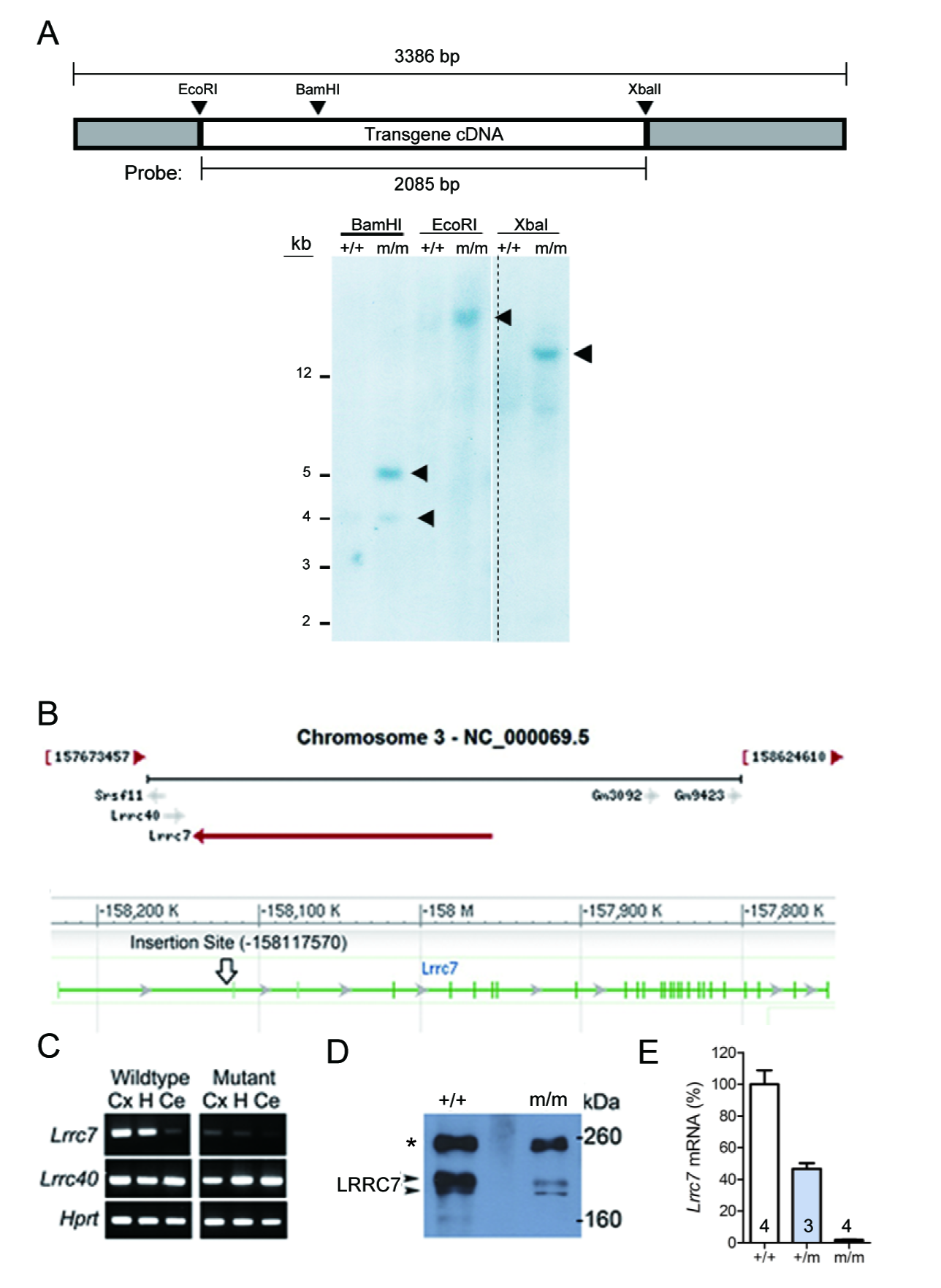
**

**Fig. S1.** Generation of *Lrrc7* mutant mice. **(A)** Diagrammatic representation of the DNA construct for oocyte injection and the probe for Southern analysis. The result suggested integration of a single copy of the transgene. **(B)** Genome view of the insertion site of the transgene at intron 1 of *Lrrc7* as determined by inverse PCR*.* **(C)** The transgene disrupted the transcription of *Lrrc7* but not the nearby gene *Lrrc40*. Cx, cortex; H, hippocampus; Ce: cerebellum. **(D)** Trace amount of LRRC7 protein was detectable in cerebral cortex of homozygotes in western blot. ***** indicates non-specific band. **(E)** Heterozygous mice expressed 46% of the normal amount of *Lrrc7* transcripts while homozygotes expressed 2% of wild-type transcripts in RT-qPCR. Same results obtained for exons 1-6 and common exons 27 – 28 for all isoforms. The latter is being shown*.* Number of mice indicated in graph.


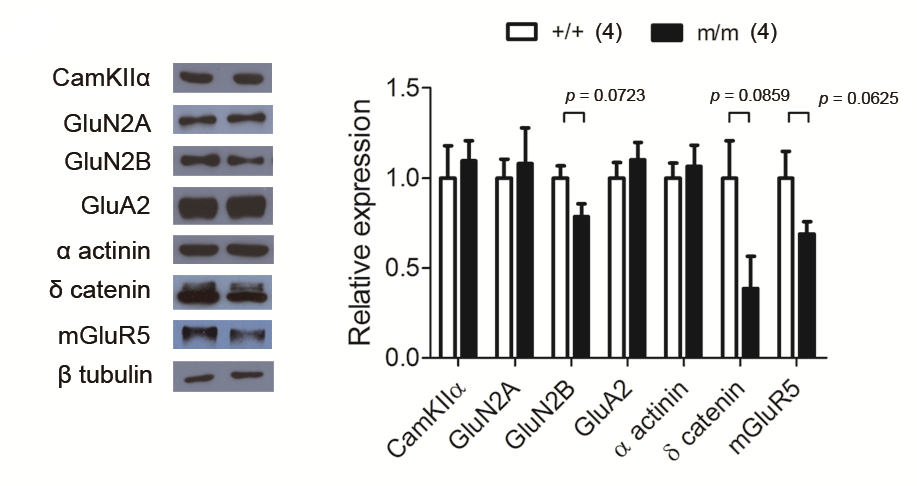


**Fig. S2.** Western analysis of synaptosome-enriched samples (P2 fraction) collected from littermate mice at postnatal day 28. Quantitation of signals showed a near significant reduction of GluN2B, δ-catenin and mGluR5 in the mutant cortex. Data are expressed as mean ± SEM. N =4 mice of each genotype (+/+, wild-type; m/m homozygous mutant), Student *t*-test**.**

Proteins were detected with antibodies for CaMKIIα (Cell Signaling, cat. 3357); GluN2A (Abcam, cat. ab14596); GluN2B (Millipore, cat. 06-600); GluA2 (Bioworld, cat. BS3658); α-actinin (Millipore, cat. MABT144); δ-catenin (Millipore, cat. 07-259); mGluR5 (Millipore, cat. AB5675); β-tubulin (Sigma, St. Louis, MO, USA, cat. T8328).


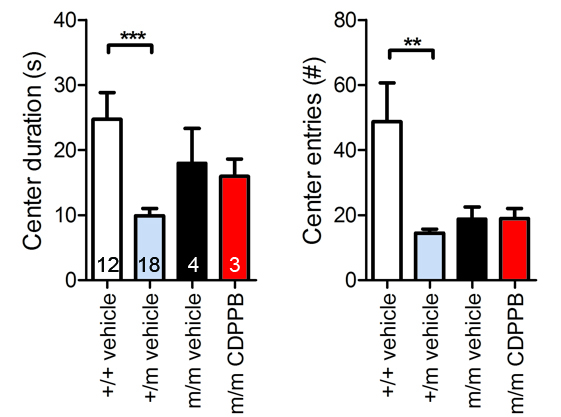


**Fig. S3.** **Heterozygous mutants stressed at the juvenile stage show anxiety-like behavior in the open filed test.** Seven litters of mice were given intraperitoneal injections from P16 to P22 as shown in Figure 2 of main text. Same mice were subjected to open field test at 8 weeks. One-way ANOVA, center duration, *P* = 0.0019; center entries, *P* = 0.0059, Tukey’s *post hoc* test, ***P* < 0.05, ****P* < 0.01. Data are expressed as mean ± SEM. Number of mice indicated in graph.
